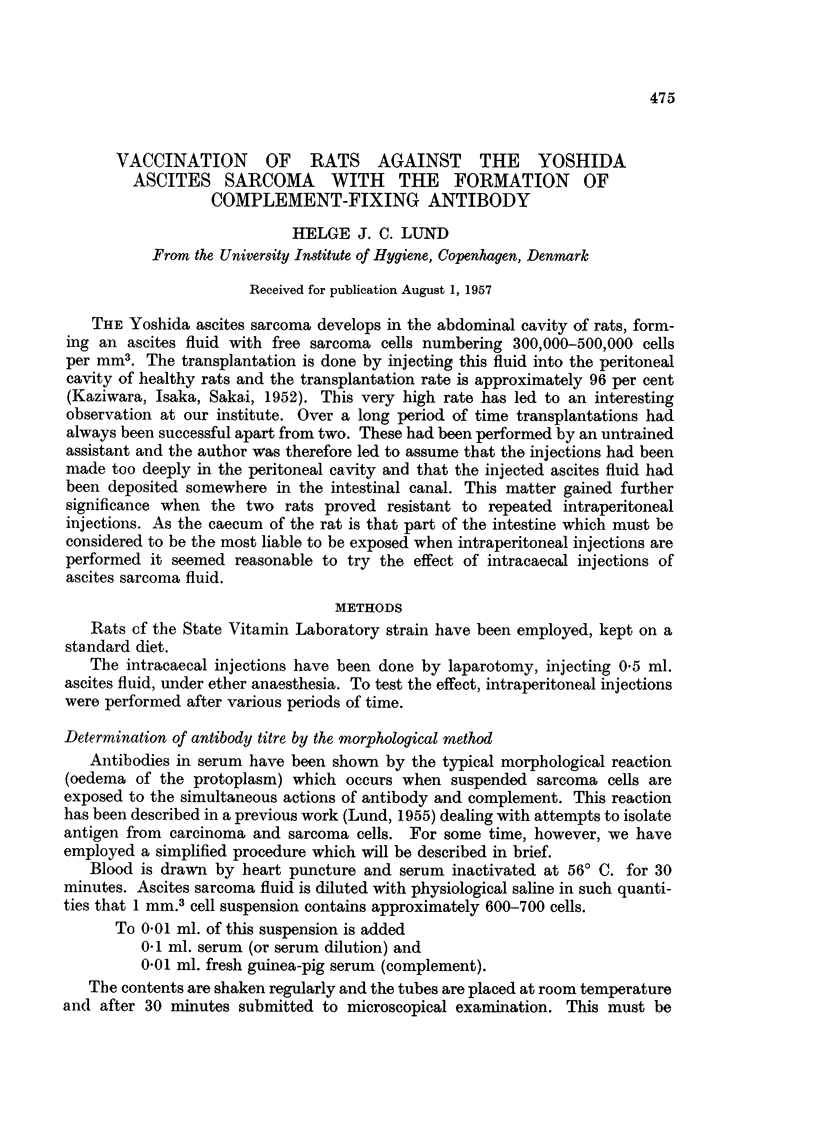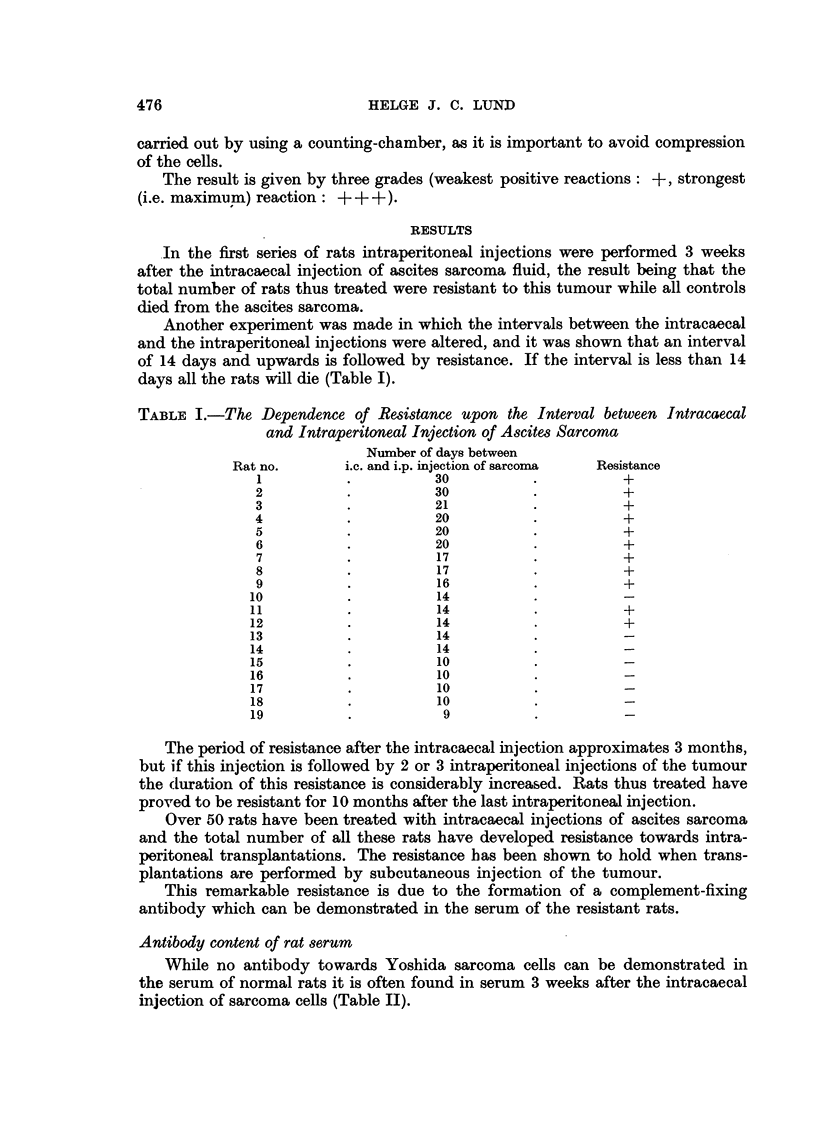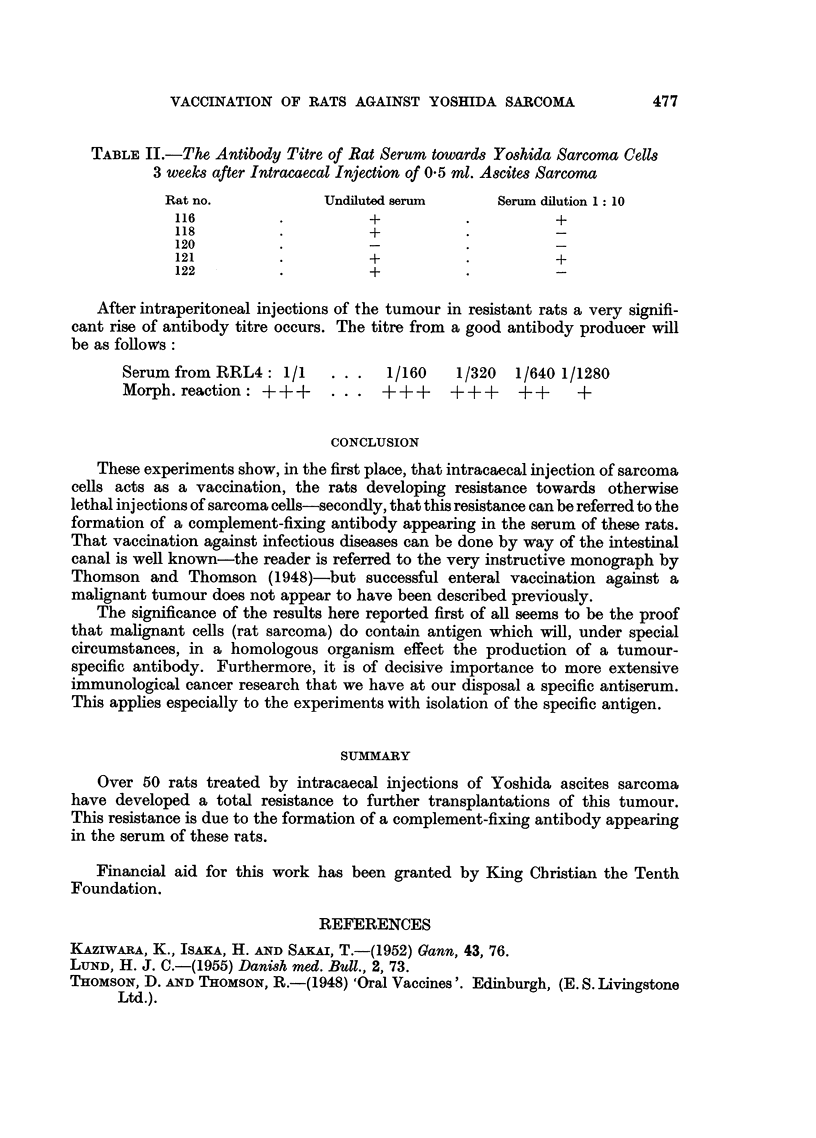# Vaccination of Rats Against the Yoshida Ascites Sarcoma with the Formation of Complement-Fixing Antibody

**DOI:** 10.1038/bjc.1957.56

**Published:** 1957-09

**Authors:** Helge J. C. Lund


### VACCINATION OF RATS AGAINST THE YOSHIDA ASCITES SARCOMA WITH THE FORMATION OF COMPLEMENT-FIXING ANTIBODY

HELGE J. C. LUND

*From the University Institute of Hygiene, Copenhagen, Denmark*

Received for publication August 1, 1957

THE Yoshida ascites sarcoma develops in the abdominal cavity of rats, forming an ascites fluid with free sarcoma cells numbering 300,000–500,000 cells per $mm^3$. The transplantation is done by injecting this fluid into the peritoneal cavity of healthy rats and the transplantation rate is approximately 96 per cent (Kaziwara, Isaka, Sakai, 1952). This very high rate has led to an interesting observation at our institute. Over a long period of time transplantations had always been successful apart from two. These had been performed by an untrained assistant and the author was therefore led to assume that the injections had been made too deeply in the peritoneal cavity and that the injected ascites fluid had been deposited somewhere in the intestinal canal. This matter gained further significance when the two rats proved resistant to repeated intraperitoneal injections. As the caecum of the rat is that part of the intestine which must be considered to be the most liable to be exposed when intraperitoneal injections are performed it seemed reasonable to try the effect of intracaecal injections of ascites sarcoma fluid.

### METHODS

Rats of the State Vitamin Laboratory strain have been employed, kept on a standard diet.

The intracaecal injections have been done by laparotomy, injecting 0·5 ml. ascites fluid, under ether anaesthesia. To test the effect, intraperitoneal injections were performed after various periods of time.

### *Determination of antibody titre by the morphological method*

Antibodies in serum have been shown by the typical morphological reaction (oedema of the protoplasm) which occurs when suspended sarcoma cells are exposed to the simultaneous actions of antibody and complement. This reaction has been described in a previous work (Lund, 1955) dealing with attempts to isolate antigen from carcinoma and sarcoma cells. For some time, however, we have employed a simplified procedure which will be described in brief.

Blood is drawn by heart puncture and serum inactivated at 56° C. for 30 minutes. Ascites sarcoma fluid is diluted with physiological saline in such quantities that 1 $mm.^3$ cell suspension contains approximately 600–700 cells.

To 0·01 ml. of this suspension is added
0·1 ml. serum (or serum dilution) and
0·01 ml. fresh guinea-pig serum (complement).

The contents are shaken regularly and the tubes are placed at room temperature and after 30 minutes submitted to microscopical examination. This must be carried out by using a counting-chamber, as it is important to avoid compression of the cells.

The result is given by three grades (weakest positive reactions : +, strongest (i.e. maximum) reaction : +++).

### RESULTS

In the first series of rats intraperitoneal injections were performed 3 weeks after the intracaecal injection of ascites sarcoma fluid, the result being that the total number of rats thus treated were resistant to this tumour while all controls died from the ascites sarcoma.

Another experiment was made in which the intervals between the intracaecal and the intraperitoneal injections were altered, and it was shown that an interval of 14 days and upwards is followed by resistance. If the interval is less than 14 days all the rats will die (Table I).

TABLE I.—*The Dependence of Resistance upon the Interval between Intracaecal and Intraperitoneal Injection of Ascites Sarcoma*

| Rat no. | Number of days between i.c. and i.p. injection of sarcoma | Resistance |
|---|---|---|
| 1 | 30 | + |
| 2 | 30 | + |
| 3 | 21 | + |
| 4 | 20 | + |
| 5 | 20 | + |
| 6 | 20 | + |
| 7 | 17 | + |
| 8 | 17 | + |
| 9 | 16 | + |
| 10 | 14 | — |
| 11 | 14 | + |
| 12 | 14 | + |
| 13 | 14 | — |
| 14 | 14 | — |
| 15 | 10 | — |
| 16 | 10 | — |
| 17 | 10 | — |
| 18 | 10 | — |
| 19 | 9 | — |

The period of resistance after the intracaecal injection approximates 3 months, but if this injection is followed by 2 or 3 intraperitoneal injections of the tumour the duration of this resistance is considerably increased. Rats thus treated have proved to be resistant for 10 months after the last intraperitoneal injection.

Over 50 rats have been treated with intracaecal injections of ascites sarcoma and the total number of all these rats have developed resistance towards intraperitoneal transplantations. The resistance has been shown to hold when transplantations are performed by subcutaneous injection of the tumour.

This remarkable resistance is due to the formation of a complement-fixing antibody which can be demonstrated in the serum of the resistant rats.

### *Antibody content of rat serum*

While no antibody towards Yoshida sarcoma cells can be demonstrated in the serum of normal rats it is often found in serum 3 weeks after the intracaecal injection of sarcoma cells (Table II).

TABLE II.—*The Antibody Titre of Rat Serum towards Yoshida Sarcoma Cells 3 weeks after Intracaecal Injection of* 0·5 *ml. Ascites Sarcoma*

| Rat no. | Undiluted serum | Serum dilution 1 : 10 |
|---|---|---|
| 116 | + | + |
| 118 | + | − |
| 120 | − | − |
| 121 | + | + |
| 122 | + | − |

After intraperitoneal injections of the tumour in resistant rats a very significant rise of antibody titre occurs. The titre from a good antibody producer will be as follows :

| | | | | | | |
|---|---|---|---|---|---|---|
| Serum from RRL4 : | 1/1 | . . . | 1/160 | 1/320 | 1/640 | 1/1280 |
| Morph. reaction : | +++ | . . . | +++ | +++ | ++ | + |

### CONCLUSION

These experiments show, in the first place, that intracaecal injection of sarcoma cells acts as a vaccination, the rats developing resistance towards otherwise lethal injections of sarcoma cells—secondly, that this resistance can be referred to the formation of a complement-fixing antibody appearing in the serum of these rats. That vaccination against infectious diseases can be done by way of the intestinal canal is well known—the reader is referred to the very instructive monograph by Thomson and Thomson (1948)—but successful enteral vaccination against a malignant tumour does not appear to have been described previously.

The significance of the results here reported first of all seems to be the proof that malignant cells (rat sarcoma) do contain antigen which will, under special circumstances, in a homologous organism effect the production of a tumour-specific antibody. Furthermore, it is of decisive importance to more extensive immunological cancer research that we have at our disposal a specific antiserum. This applies especially to the experiments with isolation of the specific antigen.

### SUMMARY

Over 50 rats treated by intracaecal injections of Yoshida ascites sarcoma have developed a total resistance to further transplantations of this tumour. This resistance is due to the formation of a complement-fixing antibody appearing in the serum of these rats.

Financial aid for this work has been granted by King Christian the Tenth Foundation.

### REFERENCES

KAZIWARA, K., ISAKA, H. AND SAKAI, T.—(1952) *Gann*, **43**, 76.
LUND, H. J. C.—(1955) *Danish med. Bull.*, **2**, 73.
THOMSON, D. AND THOMSON, R.—(1948) 'Oral Vaccines'. Edinburgh, (E. S. Livingstone Ltd.).